# Salivary extracellular vesicle-associated miRNAs as potential biomarkers in oral squamous cell carcinoma

**DOI:** 10.1186/s12885-018-4364-z

**Published:** 2018-04-18

**Authors:** Chiara Gai, Francesco Camussi, Roberto Broccoletti, Alessio Gambino, Marco Cabras, Luca Molinaro, Stefano Carossa, Giovanni Camussi, Paolo G. Arduino

**Affiliations:** 1 Department of Medical Sciences, University of Turin, C.so Dogliotti, 14 –10126 Turin, Italy; 2 Department of Surgical Sciences, University of Turin, Via Nizza 230, 10126 Turin, Italy

**Keywords:** Oral squamous cell carcinoma (OSCC), Extracellular vesicles (EVs), microRNA (miRNA), miRNA-512-3p (miR-512), miRNA-412-3p (miR-412), miR-302b-3p (miR-302b), miR-517b-3p (miR-517b), miR-27a-3p (miR-27a), miR-494

## Abstract

**Background:**

Several studies in the past have investigated the expression of micro RNAs (miRNAs) in saliva as potential biomarkers. Since miRNAs associated with extracellular vesicles (EVs) are known to be protected from enzymatic degradation, we evaluated whether salivary EVs from patients with oral squamous cell carcinoma (OSCC) were enriched with specific subsets of miRNAs.

**Methods:**

OSCC patients and controls were matched with regards to age, gender and risk factors. Total RNA was extracted from salivary EVs and the differential expression of miRNAs was evaluated by qRT-PCR array and qRT-PCR. The discrimination power of up-regulated miRNAs as biomarkers in OSCC patients versus controls was evaluated by the Receiver Operating Characteristic (ROC) curves.

**Results:**

A preliminary qRT-PCR array was performed on samples from 5 OSCC patients and 5 healthy controls whereby a subset of miRNAs were identified that were differentially expressed. On the basis of these results, a cohort of additional 16 patients and 6 controls were analyzed to further confirm the miRNAs that were up-regulated or selectively expressed in the previous pilot study. The following miRNAs: miR-302b-3p and miR-517b-3p were expressed only in EVs from OSCC patients and miR-512-3p and miR-412-3p were up-regulated in salivary EVs from OSCC patients compared to controls with the ROC curve showing a good discrimination power for OSCC diagnosis. The Kyoto Encyclopedia of Gene and Genomes (KEGG) pathway analysis suggested the possible involvement of the miRNAs identified in pathways activated in OSCC.

**Conclusions:**

In this work, we suggest that salivary EVs isolated by a simple charge-based precipitation technique can be exploited as a non-invasive source of miRNAs for OSCC diagnosis. Moreover, we have identified a subset of miRNAs selectively enriched in EVs of OSCC patients that could be potential biomarkers.

**Electronic supplementary material:**

The online version of this article (10.1186/s12885-018-4364-z) contains supplementary material, which is available to authorized users.

## Background

Oral squamous cell carcinoma (OSCC) is the most frequent cancer of the head and neck [1, 2]. Despite outstanding diagnostic and therapeutic improvements in oncology, OSCC still holds a poor prognosis with an estimated 5-year overall survival rate of 56% both in the United States and Western Europe [1, 2]. Specifically, in northern Italy, the 3-year and 5-year overall survival rate has been estimated to be 57% and 49% [3] with the latter decreasing dramatically when considering advanced or metastatic cases. Although, in recent years different biological and molecular factors have been described for the prognosis of OSCC, none of them have had a real impact on routine clinical care. Histopathological staging still remains the gold standard for post-operative management and prognosis of the disease [4]. Hence, more reliable and time saving diagnostic tools are needed.

In OSCC, metastasis spreads predominantly via a lymphatic route with cervical lymph nodes (LN) as the first location, whereas metastasis to distant sites is relatively uncommon [5]. Efficient detection and removal of LN metastasis is therefore crucial in the treatment and survival of patients with this form of carcinoma. Therefore, great expectations lie on the identification of specific predictive factors that could be used clinically for the diagnosis, prognosis and monitoring of the therapeutic response.

In the last decade, extracellular vesicles (EVs) have gained significant attention as a conceivable source of biomarkers. These small membrane-bound vesicles are categorized into 3 different types: exosomes, microvesicles or ectosomes, and apoptotic bodies [6]. EVs are secreted under different physiological and pathophysiological conditions into the extracellular milieu by a variety of cell types, including tumor cells. Tumor-derived EVs have been identified to influence the tumor environment by promoting cancer progression, survival, invasion, and angiogenesis [7]. However, as EVs carry biologically active proteins and nucleic acids from the parent cells, tumor-derived EVs could therefore act as molecular signatures of cancer cells from which they are derived [7, 8].

MicroRNAs (miRNAs) are small non-coding RNA molecules approximately 22 nucleotides in length, which act as regulatory gatekeepers of coding genes. MiRNAs are expressed in a tissue-specific manner, and changes in their expression within a tissue can be correlated with a disease status [9]. Furthermore, they can also modulate gene expression by regulating mRNA translation and/or degradation depending on complementarity between the miRNA and the mRNA [10]. MiRNAs can be secreted either through EVs and/or by forming protein-miRNA complexes with molecules such as high-density lipoproteins and AGO2, which are part of the RISC complex. However, miRNAs carried in EVs are more stable once released as the encapsulation provides protection from enzyme degradation. This therefore makes them more promising as next-generation biomarkers for cancer diagnosis and prognosis [8]. To date, cancer biomarkers carried by EVs have been studied in several types of tumors [9, 11–13], including head and neck cancer [14]. However, no work has been published on the expression of miRNAs in EVs from saliva of patients with OSCC.

The aim of the present study was therefore to evaluate whether salivary EVs of OSCC patients and healthy controls express a different pattern of miRNAs and whether the differentially expressed miRNAs could be applied as potential biomarkers for OSCC.

## Methods

### Selection of patients

The enrolled subjects were attending the Oral Medicine Section of the Department of Surgical Sciences, University of Turin, CIR-Dental School, between January and June 2015. Patients with biopsy-proven OSCC were involved in the study with the exclusion of patients with the following criteria: 1) < 18 years of age, 2) pregnant or breast feeding, 3) psychiatrically or mentally unstable. Local ethical committee approval (n° 310/2015, “A.O.U. Città della Salute e della Scienza di Torino”, Turin, Italy) was obtained and all patients provided written informed consent. Demographic information, age at the time of diagnosis and gender, smoking, tumor site, and TNM classification [15] were recorded at baseline (Table 1 and Additional file 1: Table S1). Healthy subjects presenting no clinically detectable oral lesions matched for age, gender, and risk factors were recruited as controls (Table 1).

**Table 1:** Characteristics of OSCC patients and controls enrolled in the study

Characteristics	OSCC patients	Controls	*P* value
Number	21	11	
Age	65.75 [61; 73]	61.64 [61.5; 67.5]	0.381
Range	38–78	39–75	
Gender (male/female)	12 (57%)/9 (43%)	6 (55%)/5 (45%)	
Smokers	6 (28%)	3 (27%)	

For each group, the table indicates the total number of subjects, the mean age of each group and quartiles [Q1; Q3], the minimum and maximum age of enrolled subjects, number and percentage of male and female and of smokers. The differences in ages between the two groups were not statistically significant (Student’s t test)

### Saliva collection

As previously reported [16], all subjects were asked to refrain from: eating, drinking, or oral hygiene for at least one hour prior to collection (usually between 9 and 11 a.m.); they then rinsed their mouths with water and then waited for at least 5 min before spitting into a 50 ml Falcon tube. Participants were instructed not to cough or forcefully expectorate in order to collect unstimulated saliva samples.

### HPV-16 in situ hybridization

In situ hybridization (ISH) for HPV was performed on hematoxylin and eosin sections using the Bond TM Ready-to-Use ISH HPV Probe (Leica Biosystems, Newcastle, UK) which targets the following subtypes: 16, 18, 31, 33, and 51. ISH was carried out following the manufacturer’s instructions on the automated Leica BOND system (BOND-MAX, Leica Biosystems).

### EV isolation

The sample of saliva from patients with OSCC and healthy controls was diluted 1:1 with PBS (phosphate buffered saline) and centrifuged at 3000 g for 15 min at room temperature to remove cells, debris and bacteria. The supernatant was filtered with 0.2 μm filters and transferred to a sterile tube after which, a precipitation solution (65 μL per 250 μL of saliva) was added and the mixture incubated at 4 °C overnight. The following day, samples were centrifuged at 3000 g for 30 min to precipitate EVs. The resulting supernatant was removed and samples re-centrifuged at 1500 g for 5 min to remove any remaining supernatant [17]. The pellet was resuspended in either: 100 μL of PBS for NanoSight analysis, or 100 μl of RIPA lysis buffer (Sigma Aldrich, Milan, IT) for protein extraction, or 600 μL of Lysis/Binding Buffer (mirVana Isolation Kit, Thermo Fisher Scientific, Waltham, MA, USA) and stored at − 80 °C for subsequent RNA extraction.

### EV characterization

The EV samples isolated from saliva were diluted 1:200 in physiologic solution and analyzed by NanoSight LM-10 (Malvern Instruments Ltd., Malvern, UK). The average number and size of EVs were measured by Nanoparticle Tracking Analysis (NTA) software (Malvern Instruments Ltd). Transmission electron microscopy (TEM) of negatively stained EVs (NanoVan, Nanoprobes, Yaphank, NK, USA) was also performed as described previously [17] and the images were obtained using the Joel JEM 1010 electron microscope (Jeol, Tokyo, Japan).

### Western blot analysis

Protein concentration in EV samples was measured by Bradford assay. Protein samples were loaded on polyacrylamide gel at the concentration of 30 μg/well and separated by SDS/PAGE, using 4–15% precast gel (Mini-PROTEAN® Precast Gels, Bio-Rad, Hercules, CA, USA). Proteins were transferred on nitrocellulose membranes by liquid electrophoresis. Membranes were immunoblotted by polyclonal antibodies anti-CD9, CD63, TSG101, and Alix (Santa Cruz Biotechnologies, Dalls, TX, USA). Protein-bands were detected by chemiluminescent Clarity™ ECL Western Blotting Substrate (Bio-Rad) and analyzed by ChemiDoc™ XRS + System (Bio-Rad).

### RNA extraction and quantification

miRNAs were extracted from purified EVs by mirVana Isolation Kit (Thermo Fisher Scientific), according to the manufacturer’s instruction. RNA concentration was measured by Nanodrop ND-1000 (Thermo Fisher Scientific), and the ratio 260/280 and 260/230 showed no contaminations. RNA integrity was assessed by a Bioanalyzer (Agilent, Santa Clara, CA) using the RNA 6000 Pico Kit (Agilent).

### miRNA expression analysis by qRT-PCR array

To select differentially expressed miRNAs, qRT-PCR array analysis was performed on EVs isolated from five patients with OSCC and five healthy controls. The concentration of selected RNA samples was up to 20 ng/μl and 50 ng of total RNA were retro-transcribed to cDNA with TaqMan® MicroRNA Reverse Transcription Kit (Thermo Fisher Scientific). cDNA was pre-amplified with Megaplex™ RT Primers, Human Pool Set v3.0 and TaqMan® PreAmp Master Mix (Thermo Fisher Scientific) using a Veriti Thermal Cycler (Thermo Fisher Scientific). The expression profile of a panel of 754 human microRNAs was evaluated by a TaqMan® Array Human MicroRNA Card Set v3.0 (Thermo Fisher Scientific) using the real-time thermal cycler 7900HT (Thermo Fisher Scientific).

### qRT-PCR

On the basis of results obtained from the array, we studied miRNAs up-regulated or selectively expressed by patients in a cohort of additional 16 OSCC patients and 6 controls. 500 ng of total RNA was retro-transcribed to cDNA with miScript II RT Kit (Qiagen, Hilden, D) and evaluated for the expression of five miRNAs up-regulated in patients compared to healthy controls (miR-412-3p, miR-489-3p, miR-512-3p, miR-597-5p, and miR-603). Furthermore, eight miRNAs exclusively expressed only in OSCC patients (miR-27a-3p, miR-302b-3p, miR-337-5p, miR-373-3p, miR-494-3p, miR-517b, and miR-520d-3p, miR-645) were also evaluated. Each sample was run in triplicate and each miRNA-specific primer was run in a separate reaction. SnoRNA RNU6B and miR-191 were used as endogenous control as previously described [18–20] due to their stable expression in saliva samples which was also confirmed in the current study by qRT-PCR in tested salivary EV samples.

The qRT-PCR reaction mix was composed of 2 ng of cDNA, 100 nM miScript Universal Primer (Qiagen), 100 nM miRNA-specific primer (Eurofins Genomics, Ebersberg, D), 5 μl QuantiTect SYBR Green PCR Master Mix (Qiagen), and nuclease free water (Qiagen) to reach a final reaction volume of 10 μl. The Real-Time Thermal Cycler Quant Studio 12 k (Thermo Fisher Scientific) was used for analysis.

### Enrichment analysis

Enrichment analysis for biological pathways was performed for miRNAs that were found to be up-regulated (*p* < 0.09) or only expressed by OSCC patients. Kyoto Encyclopedia of Gene and Genomes (KEGG) pathway analysis was performed through the DIANA-mirPath v.3.0 [21] online software and miRNA targets were searched on microT-CDS [22]. Results were merged by a “pathway-union” criterion. The *p* value was calculated by DIANA software online with: False Discovery Rate (FDR) correction, p value threshold at 0.05 and MicroT threshold at 0.8. Fisher’s exact test was used as the statistical method for the enrichment analysis.

### Discrimination power analysis

The discrimination power of the up-regulated miRNAs as biomarkers for OSCC diagnosis was evaluated by the Receiver Operating Characteristic (ROC) curves [23]. The ROC curves were constructed using the relative quantification (RQ) of the expression levels of controls and OSCC patients by the demo version of GraphPad Prism 6.01 software. Sensitivity, specificity, area under curve (AUC) and p value were calculated by the software. The optimal threshold value was decided using Youden’s index (sensitivity + specificity-1) [24].

### Statistical analysis

For array data analysis, SDS Software v.2.3 (Thermo Fisher Scientific) was used to calculate Raw Ct values, with an automatic baseline and threshold. ExpressionSuite Software 1.1 (Thermo Fisher Scientific) was used to calculate RQ (2^-∆∆Ct^) values. Data were normalized using global normalization, an algorithm that finds the assays common to every sample and then uses the median Ct of those assays as the normalization factor, on a per sample basis [25]. Ct values > 35 or with Amp score < 0.7 were excluded from the analysis. To identify candidate miRNAs differentially expressed between patients and controls, we selected miRNAs with low *p* values (*p* ≤ 0.05). *P* values were calculated by the ExpressionSuite software using Student’s t-test for sample group comparisons, without multiple test correction. Further to this, we screened the group for miRNAs expressed in every sample and with low variability among the same group.

For qRT-PCR data analysis, Excel software (Microsoft Office 365 ProPlus) was used to calculate ∆Ct, −∆∆Ct, and RQ for patients and controls. Statistical analysis was performed on RQ values through the demo version of GraphPad Prism 6.01 software using an unpaired non-parametric two-sided Mann-Whitney test. Confidence level was set at 95% (*p* value ≤0.05).

## Results

### Patient characterization

A total of 21 patients with OSCC (12 men and 9 women) were analyzed (Table 1). The TNM staging system identified the following lesion categories: T1 (*n* = 7), T2 (*n* = 8), T3 (*n* = 3), T4 (*n* = 3); according to the histology, of biopsy specimens, three patients were identified as well differentiated, twelve as moderately differentiated and six as poorly differentiated. The lateral border of the tongue was the most commonly affected site (24%), followed by the floor of the mouth and gingiva (19% respectively), the palate (14%), the pelvis (9.5%) and lastly other sites (19%) (Additional file 1: Table S1). Five, out of 21 patients, were positive for HPV (23.8%). A total of 11 controls (seven men and four women) were also analyzed. The subjects did not show oral lesions, infections, or tumor history. Controls and OSCC patients were matched based on gender, age and risk factor, as shown in Table 1.

### EV characterization

**Fig. 1:**
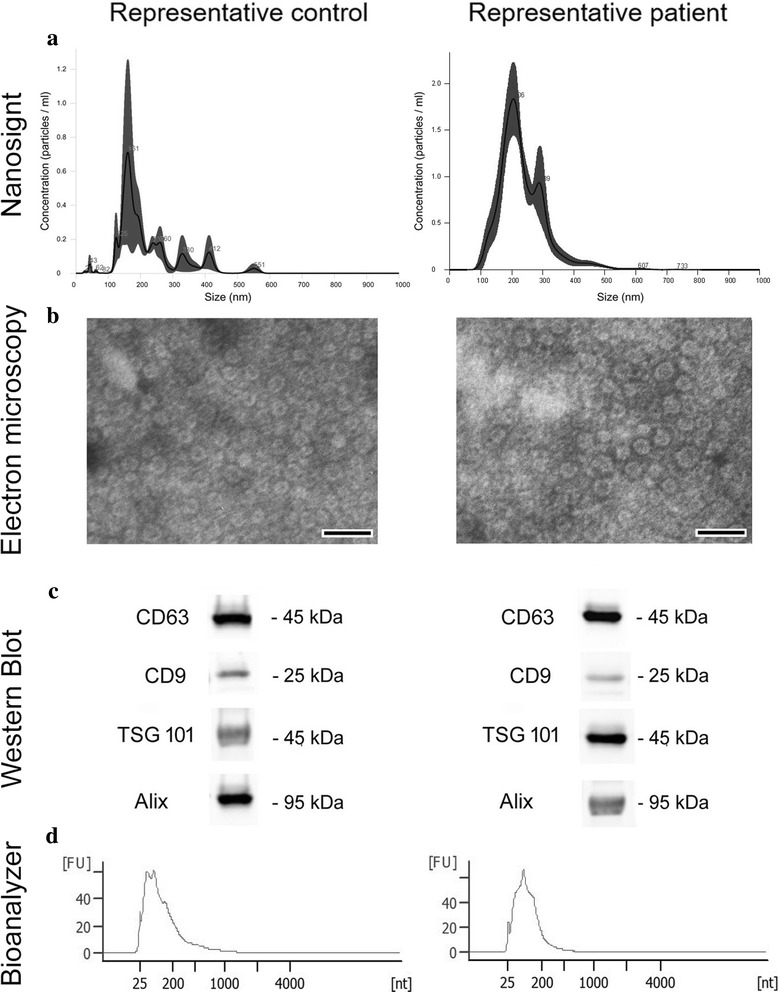
Characterization of salivary EVs. (**a**) Representative NanoSight image of isolated EVs showing particle size (nm)/concentration (10^8 particles/ml) of a representative control (left) and a representative OSCC patient (right). (**b**) Representative transmission electron microscopy image of purified EVs negatively stained with NanoVan (JEOL Jem-1010 electron microscope, black line = 200 nm) of a control (left) and a patient (right). (**c**) Representative western blots confirming the expression of the exosome markers: CD63, CD9, Tsg101, and Alix, on salivary EVs from a control (left) and a OSCC patient (right). (**d**) Representative profiles of RNA isolated from EVs of a healthy control (left) and a patient (right). The graphs show fluorescence intensity [FU]/nucleotide length [nt] and were obtained through bioanalyzer analysis. Four experiments were performed with similar results

According to NanoSight results, EVs isolated from saliva samples through charge-based precipitation appeared as a heterogeneous population with a size ranging from 100 to 300 nm (Fig. 1a). TEM analysis confirmed the characteristic shape, aspects, and dimensions of EVs (Fig. 1b). Furthermore, we observed that the size and concentration of salivary EVs from OSCC patients were slightly increased compared to healthy controls, however, the differences were not statistically significant. Western Blot analysis demonstrated the expression of the typical exosome markers: CD63, CD9, TSG 101, and Alix (Fig. 1c). Additionally, Western blot analysis performed in duplicate on samples from 10 OSCC patients and 8 controls was also positive for the exosome markers: CD63 and TSG 101 marker, confirming the reproducibility of the isolation method (Additional file 2: Figure S1). The expression of the exosome markers was similar for both OSCC patients and controls. According to Bioanalyzer results, RNA cargo is mainly constituted of RNAs ranging from 20 to 200 nucleotides, whereas the ribosomal RNAs 18 s and 28 s were absent (Figure 1d). No differences were observed between RNA profiles of OSCC patients and controls (Additional file 3: Figure S2).

### miRNA expression analysis by qRT-PCR array

On analyzing the expression of miRNAs in salivary EVs, we identified five miRNAs to be up-regulated (miR-412-3p, miR-489-3p, miR-512-3p, miR-597-5p, and miR-603), and six miRNAs down-regulated (miR-193b-3p, miR-30e-3p, miR-376c-3p, miR-484, miR-720, and miR-93-3p) in tumor EVs compared to controls (Table 2). Moreover, eight miRNAs were exclusively detected in EVs from OSCC patients, while 14 miRNAs were specific only to EVs from controls (Table 3). The complete qRT-PCR array results are shown in Additional file 4: Table S2, reporting all miRNAs expressed in EVs of both groups of patients.

**Table 2:** miRNAs differentially expressed in salivary EVs of patients with OSCC compared to healthy controls

	miRNA	RQ	RQ Min	RQ Max	*P* value
UP	miR-412-3p	9.404	5.855	15.104	0.007
	miR-489-3p	35.07	17.47	70.41	0.020
	miR-512-3p	5.13	2.05	12.85	0.031
	miR-597-5p	3.62	1.83	7.14	0.026
	miR-603	2.36	1.30	4.28	0.042
DOWN	miR-193b-3p	0.26	0.05	1.27	0.042
	miR-30e-3p	0.21	0.07	0.65	0.010
	miR-376c-3p	0.17	0.08	0.32	0.035
	miR-484	0.44	0.23	0.85	0.048
	miR-720	0.29	0.09	0.96	0.017
	miR-93-3p	0.39	0.15	1.05	0.044

MiRNAs were considered up-regulated (UP) or down-regulated (DOWN) for *p* value < 0.05 and similar expression levels in each sample. Relative quantification (RQ) = 2^-∆∆Ct^

**Table 3:** miRNAs exclusively expressed in salivary EVs of the OSCC patients or the controls

	miRNA	Ct mean	St dev
Only patients	miR-27a-3p	29.5	1.33
	miR-302b-3p	33.9	0.34
	miR-337-5p	30.0	1.32
	miR-373-3p	30.9	0.40
	miR-494-3p	33.5	1.24
	miR-517b	32.2	1.57
	miR-520d-3p	31.2	1.42
	miR-645	34.2	0.40
Only controls	miR-126-5p	32.6	1.86
	miR-127-3p	32.1	1.39
	miR-1276	31.4	0.95
	miR-1289	33.8	1.34
	miR-144-5p	32.4	2.55
	miR-182-5p	32.2	1.11
	miR-30d-3p	31.3	1.22
	miR-520c-3p	32.1	1.70
	miR-550a-5p	32.1	0.29
	miR-628-3p	30.5	0.70
	miR-944	32.9	0.93
	miR-99a-3p	31.1	1.21
	miR-942	31.1	0.85
	RNU48	32.1	1.03

MiRNAs were considered as expressed only by patients or controls when at least 3 samples out of 5 have Ct > 35 or show no expression

### qRT-PCR expression analysis

**Fig. 2:**
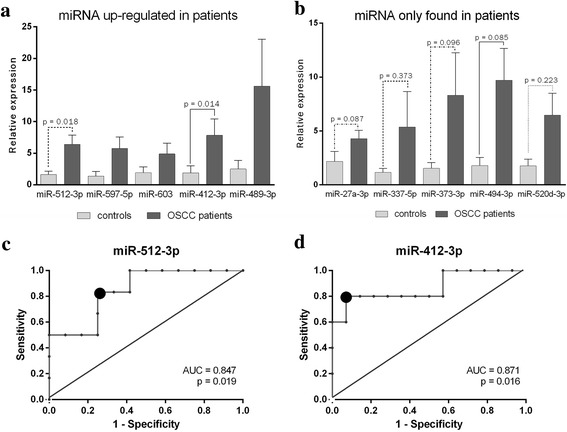
miRNA relative expression detected by qRT-PCR in salivary EV samples from OSCC patients compared to normal subjects. (**a**) Expression levels of miRNAs that were significantly up-regulated in patients. (**b**) Expression levels of miRNAs that were exclusively expressed by OSCC patients. The bars represent mean relative expression (2^-∆∆Ct) of control and patient groups ± SEM, *p* value (two-sided Mann-Whitney test) are reported. ROC curve describing predictive potency of the up-regulated miRNAs as a diagnostic test. The curves represent specificity versus sensitivity of miR-512-3p (**c**), miR-412-3p (**d**). Data are derived from miRNAs’ expression levels (RQ) of OSCC patients and controls. The big gray dots indicate the optimal threshold value of sensitivity and specificity determined by the maximum Youden’s index (sensitivity+specificity-1)

After an initial screening by qRT-PCR array, we selected 11 miRNAs for subsequent analysis. We chose the five miRNAs up-regulated in OSCC patients (miR-412-3p, miR-489-3p, miR-512-3p, miR-597-5p, and miR-603) and eight miRNAs expressed only by OSCC patients (miR-27a-3p, miR-302b-3p, miR-337-5p, miR-373-3p, miR-494-3p, miR-517b, and miR-520d-3p, miR-645). The analysis of the up-regulated miRNAs showed a significant up-regulation of miR-412-3p and miR-512-3p in OSCC patients with respect to controls (Fig. 2a). The qRT-PCR analysis of miRNAs detected only in OSCC patients through qRT-PCR array showed that miR-27a-3p, miR-337-5p, miR-373-3p, miR-494-3p, and miR-520d-3p were overexpressed in patients however still present in controls (Fig. 2b). MiR-27a-3p, miR-373-3p and miR-494-3p showed a *p* value lower than 0.1, indicating a conserved but not statistically significant trend of up-regulation in OSCC patients (Fig. 2b). MiR-302b-3p and miR-517b-3p were confirmed to be expressed only in patients (data not shown), while miR-645 expression level was comparable to controls (data not shown).

### Discrimination power of miRNAs as OSCC biomarkers

ROC curves were constructed to evaluate the discrimination power of the two up-regulated miRNAs as potential biomarkers for OSCC diagnosis. For each miRNA, the ROC curves express the sensitivity (true positive rate) versus 1-specificity (false positive rate) at various cut-off values, the AUC, indicating the discrimination power of the biomarker, and the *p* value (Fig. 2c, d). The optimal threshold value was set as the maximum Youden’s index (sensitivity + specificity-1) represented as a black circle. MiR-512-3p (Fig. 2c) and miR-412-3p (Fig. 2d) showed high sensitivity and specificity, with high AUC values of 0.847 and 0.871 respectively, and *p* values lower than 0.02.

### KEGG pathway enrichment analysis

**Fig. 3:**
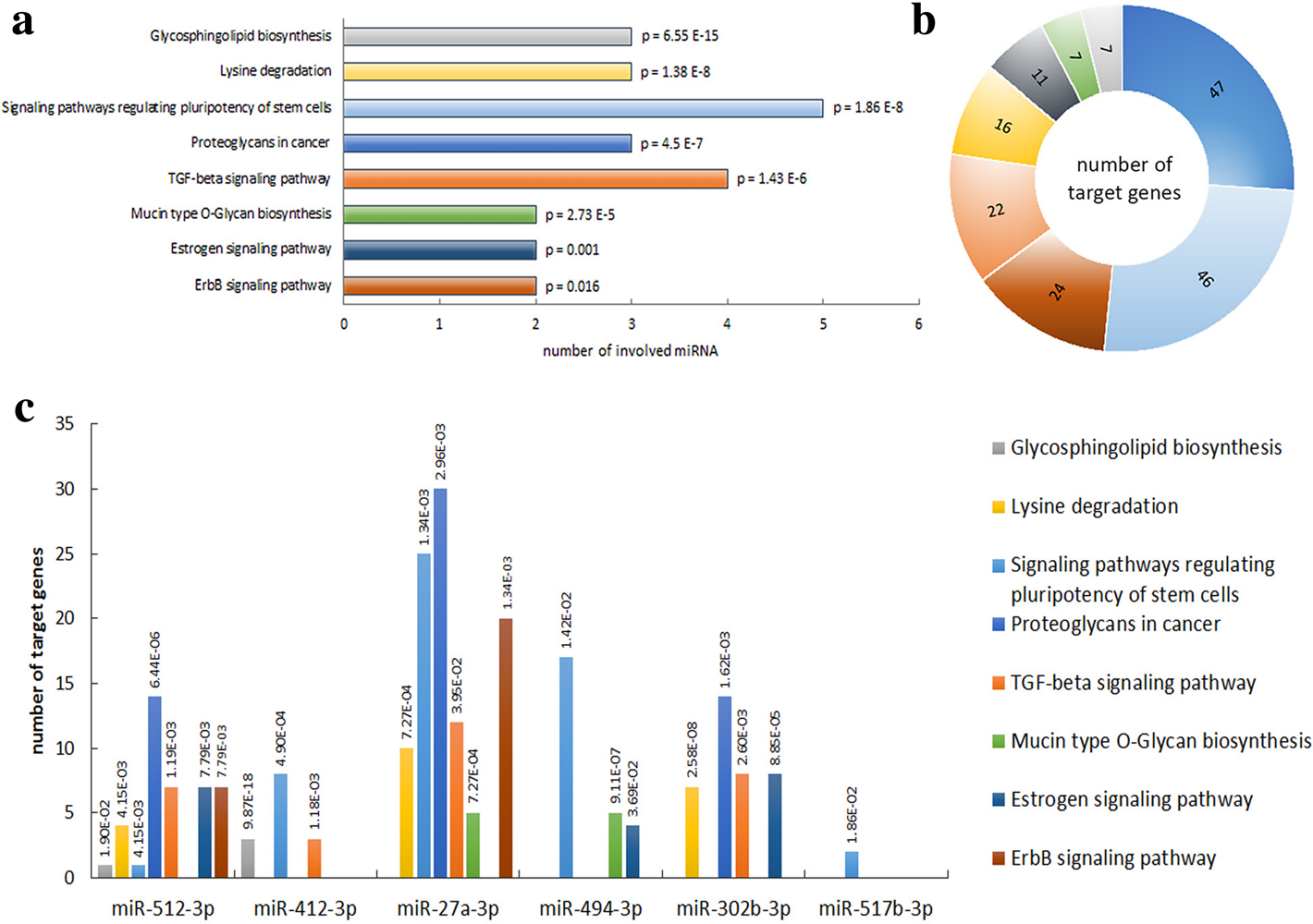
KEGG pathway enrichment analysis for up-regulated miRNAs (miR-512-3p, miR-412-3p, miR-27a-3p, miR-494-3p) or miRNAs only expressed in salivary EV from OSCC patients (miR-302b-3p, miR-517b-3p). (**a**) The graph shows significantly enriched biological pathways labeled with their respective *p* values. X axis shows the number of miRNAs involved in each pathway. (**b**) The graph shows the number of target genes for each enriched pathway. (**c**) The bars represent the number of target genes for each miRNA in each pathway and the respective *p* value. The legend (lower-right) shows the color assigned to each pathway and is valid for all the figures (**a**-**c**). The p value was calculated with FDR correction and threshold was set as 0.05

The four miRNAs (miR-512-3p, miR-412-3p, miR-27a-3p, and miR-494-3p) confirmed to be up-regulated and the two miRNAs (miR-302b-3p and miR-517b-3p) confirmed to be expressed only in OSCC patients by qRT-PCR were selected for KEGG pathway enrichment analysis. Eight pathways were found to be significantly enriched for at least two of the tested miRNAs (Fig. 3a). Furthermore, Fig. 3b shows the number of predicted target genes involved in each pathway and Fig. 3c shows, for each miRNA, the number of predicted target genes in each pathway and the respective p value.

## Discussion

The use of saliva as a diagnostic biofluid has been widely recognized, and it has many advantages over other specimens like blood, exfoliated cells and urine [26, 27]. Salivary biomarkers have the potential to serve as non-invasive, widely accessible screening tools. In fact, the collection is inexpensive and can be easily performed. Identifying the proper salivary biomarker profile could contribute to the current screening method of oral cancer, which is limited to physical exam and biopsy of suspicious lesions [26].

Several works describe the possibility to detect RNA biomarkers of numerous diseases in saliva [26–28] and, more specifically, miRNAs associated with oral cancer [26–31]. Evidence demonstrates that it is possible to isolate EV-associated RNA from saliva and oral samples [28, 32, 33]. However, to date, miRNA expression analysis in EVs from OSCC have never been reported.

In this work, according to previous evidence [17, 32–35], we successfully isolated EVs from saliva. A previous study [17] showed that most of the salivary RNA was associated with EVs. Through Bioanalyzer RNA profiles, we observed that EVs were enriched with RNAs ranging from 20 to 200 nucleotides whereas ribosomal RNAs were nearly absent. Our results are in accordance with other published data of EV RNA cargo [33, 36, 37]. Molecular analysis of miRNAs revealed an up-regulation of miR-412-3p, miR-512-3p, miR-27a-3p, miR-373-3p, miR-494-3p in salivary EVs from OSCC patients. Furthermore, we found that miR-302b-3p and miR-517b-3p were expressed specifically only in samples from the OSCC group. KEGG pathway enrichment analysis based on predicted miRNA targets provides speculative information of miRNA functions. Eight pathways showed a statistically significant enrichment with each pathway predicted to involve two or more miRNAs. For instance, miR-512-3p and miR-27a-3p could target respectively 7 and 20 genes involved in the ErbB signaling pathway. The pathway is known to promote cell proliferation and survival in several solid tumors [38] and has been shown to be activated in OSCC as well [39–41]. MiR-512-3p, miR-27a-3p, and miR-302b-3p could target respectively 14, 30, and 14 genes regulating proteoglycan in cancer pathways. Evidence has shown that CD44, which can be targeted by both miR-512-3p and miR-302b-3p, and the downstream pathway promote cell invasion and migration upon c-Fos stimulation in OSCC [42]. Increased CD44 expression has been associated with ERK1/2 phosphorylation, and increased tumor aggressiveness [43]. Moreover, high CD44 levels have been described as a characteristic feature of cancer stem-like cells in OSCC [44]. In addition, miR-512-3p, miR-412-3p, miR-27a-3p, and miR-302b-3p could target several genes of the TGFβ signaling pathway, including TGFβR2 gene. Interestingly, it has been previously reported that TGFβR2 is commonly reduced in oral epithelium and stroma in OSCC patients [45]. In line with cancer stem like cells, 46 genes involved in signaling pathways regulating pluripotency of stem cells can also be targeted by miR-512-3p, miR-27a-3p, miR-494-3p, miR-517b-3p, and miR-412-3p. The overexpression of Bmi1, which can be targeted by miR-494 and miR-27a-3p, has been shown to promote formation, growth, migration, and metastasis in a subpopulation of tumor cells of the HNSCC [46]. Basing on these observations, we speculate that the increase of miRNA that target genes involved in tumor progression in salivary EVs might represent a defenses mechanism of tumor cells to eliminate anti-tumor miRNAs. However, due to the speculative nature of this analysis, the relation between miRNAs and target genes and pathways should be further proven experimentally.

To better evaluate the discrimination power of the up-regulated miRNAs as OSCC biomarkers, we constructed ROC curves. MiR-512-3p and miR-412-3p resulted to be either sensitive and specific, as shown by high AUC values (0.847 and 0.871 respectively, with *p* values < 0.02) and maximum Youden’s Index. This indicates that the two miRNAs are good predictors and can be suggested as new candidate biomarkers for OSCC, which can be evaluated through further studies on a larger population. On the other hand, the up-regulation of miR-27a-3p and miR-494-3p can be used as indicators, but are not sufficient as diagnostic biomarkers. Nevertheless, the involvement of miR-494-3p and miR-27a-3p in OSCC is supported by the literature. MiR-494 has been previously isolated from blood of OSCC patients and proposed as a biomarker [47]. MiR-27a-3p is involved in the progression of OSCC by targeting YAP1 and therefore inhibiting epithelial to mesenchymal transition processes [48]. Furthermore, miR-27a-3p can also target MCPH1, which acts as an onco-suppressor gene [49]. It has also been proposed that high levels of miR-27a increase heat sensitivity in OSCC cells, enhancing hyperthermia-induced-cell death [50]. Moreover, miR-27a-3p seems to play a role in progression and metastasis of nasopharyngeal carcinoma [51], gastric cancer [52], esophageal cancer [53], and has been proposed as an EV-associated biomarker for colorectal cancer [54]. No evidence has been reported confirming the involvement of miR-412-3p, miR-512-3p, miR-302b-3p, and miR-517b-3p in OSCC, thus our work provides new insights about the dysregulation of miRNAs in the tumor environment. It is worth mentioning that miR-512-3p has been reported to be up-regulated in metastatic prostate cancer [55], and conversely shown anti-tumor activity in non-small cell lung cancer [56] and hepatocellular carcinoma [57].

On the other hand, our molecular analysis revealed the expression, although not statistically significant, of several miRNAs that have been reported in the literature as up-regulated in whole saliva or plasma of OSCC patients and have been proposed as biomarkers. For example, we observed the expression of both miR-31-5p and miR-31-3p in OSCC patients, however the levels were comparable with controls (Additional file 4: Table S2). Several studies have reported that miR-31 is overexpressed and/or involved in OSCC [58–63]. MiR-184, expressed in OSCC patients and controls, has been reported to have a good diagnostic value [30]. MiR-708 is up-regulated in progressing oral premalignant lesions [29] and was increased but without any statistically significant up-regulation in our group of patients (Additional file 4: Table S2). The discrepancies among the miRNAs up-regulated in our study and in other studies in the literature may be due to the relatively small number of recruited patients and controls and to the different geographical origin of the group of cases enrolled in each study. Moreover, most of the studies detected miRNAs in whole saliva [18, 20, 29, 30] or plasma [58], instead of salivary EVs. This may lead to different results since miRNAs can be differentially represented in whole saliva and salivary EVs, as it has been previously described for total plasma or plasma-derived EVs [64, 65].

## Conclusions

In this work, we demonstrated the possibility to use salivary EVs as a non-invasive source of miRNAs for OSCC diagnosis and we identified two miRNAs (miR-412-3p and miR-512-3p) overexpressed in OSCC patients and two miRNAs (miR-302b-3p and miR-517b-3p) selectively enriched in EVs from OSCC patients. These four miRNAs have the potential to be used as biomarkers.

## Additional files

Additional file 1: **Table S1.** Characteristics and tumor staging of OSCC patients enrolled in the study. (DOCX 16 kb)

Additional file 2: **Figure S1.** Protein expression analysis of exosome markers CD63 and TSG101. For each protein, the picture shows two western blot experiments performed on salivary EVs from 10 OSCC patients (left) and 8 controls (right). (TIF 373 kb)

Additional file 3: **Figure S2.** Bioanalyzer RNA profiles. Profiles of RNA isolated from salivary EVs of four controls (left) and four patients (right) in duplicate (a, b). The graphs show fluorescence intensity [FU]/nucleotide length [nt] and were obtained by bioanalyzer analysis. (TIF 1968 kb)

Additional file 4: **Table S2.** miRNAs expressed in both controls and OSCC patients. (DOCX 44 kb)

## Abbreviations

AGO2: protein argonaute-2
EVs: Extracellular vesicles
HNSCC: Head and neck squamous-cell carcinoma
KEGG: Kyoto Encyclopedia of Gene and Genomes
LN: lymph node
miRNA: microRNA
OSCC: Oral squamous cell carcinoma
RISC: RNA-induced silencing complex
ROC: Receiver Operating Characteristic
RQ: Relative quantification
TEM: Transmission electron microscopy
