# Supplementary material for: Salivary extracellular vesicle-associated miRNAs as potential biomarkers in oral squamous cell carcinoma
Source: BMC Cancer. 2018 Apr 18;18:439. doi: 10.1186/s12885-018-4364-z (PMC5907383; doi:10.1186/s12885-018-4364-z)
Supplement: Supplementary file 1 — Table S1. Characteristics and tumor staging of OSCC patients enrolled in the study. (DOCX 16 kb) [file 12885_2018_4364_MOESM1_ESM.docx]

**Table S1**

| **OSCC patient** | **T** | **N** | **M** | **G** | **Smoke** | **Oral site of involvement** |
| --- | --- | --- | --- | --- | --- | --- |
| **#1** | 2 | 1 | 0 | 3 | No | Palate |
| **#2** | 3 | 1 | 0 | 3 | No | Palate |
| **#3** | 2 | 0 | 0 | 2 | Yes | Palate |
| **#4** | 2 | 0 | 0 | 3 | No | Floor of the mouth |
| **#5** | 2 | 1 | 0 | 2 | No | Lateral border of the tongue |
| **#6** | 4 | 1 | 0 | 3 | No | Gingiva |
| **#7** | 2 | 1 | 0 | 2 | Yes | Floor of the mouth |
| **#8** | 1 | 0 | 0 | 1 | No | Lateral border of the tongue |
| **#9** | 2 | 0 | 0 | 2 | Yes | Lateral border of the tongue |
| **#10** | 1 | 0 | 0 | 2 | No | Floor of the mouth |
| **#11** | 3 | 2 | 0 | 2 | Yes | Floor of the mouth |
| **#12** | 1 | 0 | 0 | 2 | No | Gingiva |
| **#13** | 2 | 1 | 0 | 2 | Yes | Gingiva |
| **#14** | 1 | 0 | 0 | 1 | No | Lower lip |
| **#15** | 3 | 2 | 0 | 2 | No | Lateral border of the tongue |
| **#16** | 1 | 2 | 0 | 2 | No | Lateral border of the tongue |
| **#17** | 2 | 0 | 0 | 3 | No | Pelvis |
| **#18** | 4 | 0 | 0 | 2 | No | Buccal mucosa |
| **#19** | 4 | 0 | 0 | 2 | No | Maxillary tuber |
| **#20** | 1 | 0 | 0 | 1 | No | Gingiva |
| **#21** | 1 | 0 | 0 | 3 | Yes | Pelvis |

**Table S1:** **Demographic characteristics and tumor staging of OSCC patients enrolled in the study.**
