# Supplementary figures and images for: Salivary extracellular vesicle-associated miRNAs as potential biomarkers in oral squamous cell carcinoma
Source: BMC Cancer. 2018 Apr 18;18:439. doi: 10.1186/s12885-018-4364-z (PMC5907383; doi:10.1186/s12885-018-4364-z)

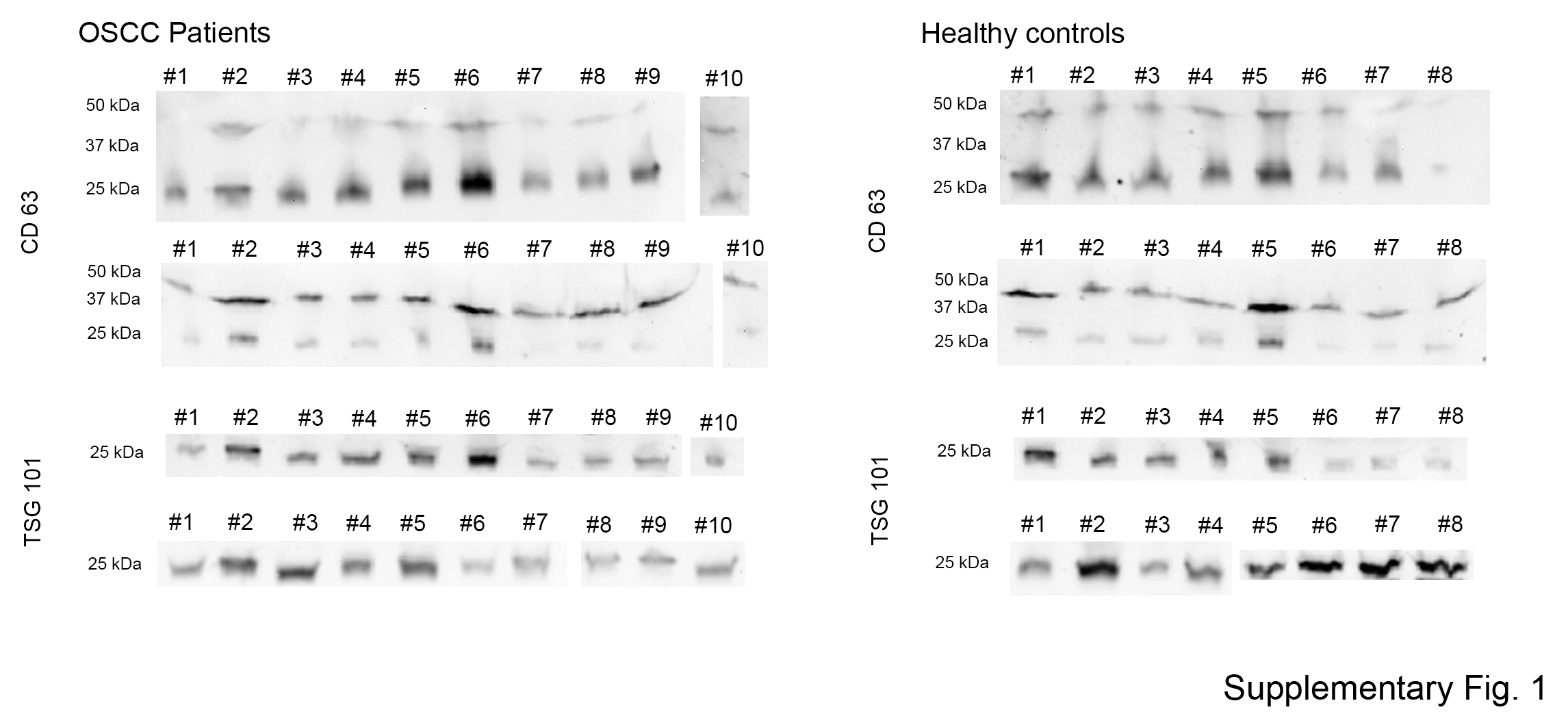

Supplement: Supplementary file 2 — Figure S1. Protein expression analysis of exosome markers CD63 and TSG101. For each protein, the picture shows two western blot experiments performed on salivary EVs from 10 OSCC patients (left) and 8 controls (right). (TIF 373 kb) [file 12885_2018_4364_MOESM2_ESM.tif]

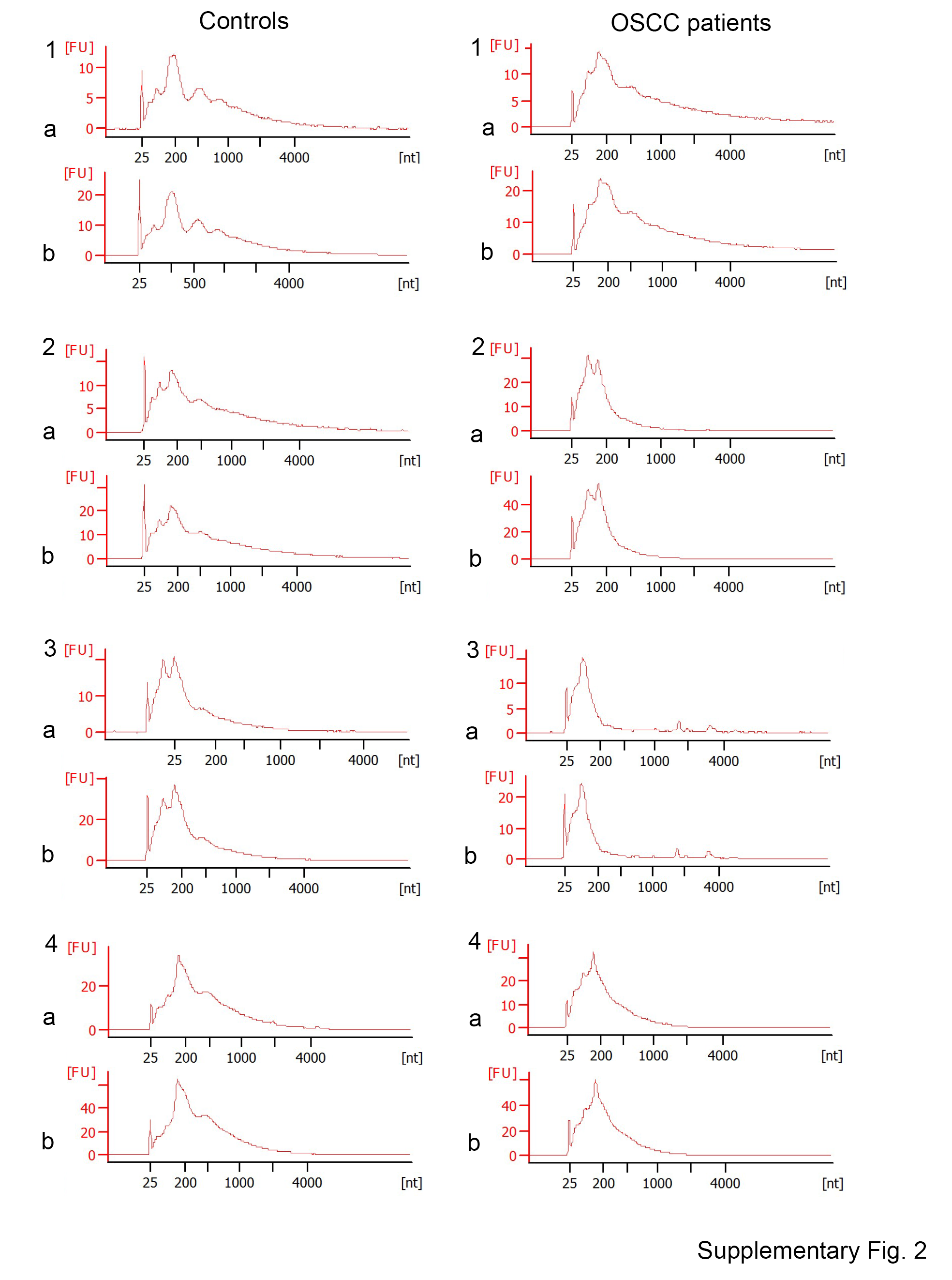

Supplement: Supplementary file 3 — Figure S2. Bioanalyzer RNA profiles. Profiles of RNA isolated from salivary EVs of four controls (left) and four patients (right) in duplicate (a, b). The graphs show fluorescence intensity [FU]/nucleotide length [nt] and were obtained by bioanalyzer analysis. (TIF 1968 kb) [file 12885_2018_4364_MOESM3_ESM.tif]
