# Supplementary material for: Salivary extracellular vesicle-associated miRNAs as potential biomarkers in oral squamous cell carcinoma
Source: BMC Cancer. 2018 Apr 18;18:439. doi: 10.1186/s12885-018-4364-z (PMC5907383; doi:10.1186/s12885-018-4364-z)
Supplement: Supplementary file 4 — Table S2. miRNAs expressed in both controls and OSCC patients. (DOCX 44 kb) [file 12885_2018_4364_MOESM4_ESM.docx]

**Table S2**

|  | **Controls** | | | | **OSCC patients** | | | |
| --- | --- | --- | --- | --- | --- | --- | --- | --- |
| **miRNA** | **Cт Mean** | **RQ** | **RQ Min** | **RQ Max** | **Cт Mean** | **RQ** | **RQ Min** | **RQ Max** |
| let-7a-5p | 27.6 | 1.0 | 0.59 | 1.68 | 28.5 | 0.68 | 0.13 | 3.48 |
| let-7b-5p | 25.8 | 1.0 | 0.73 | 1.38 | 27.6 | 0.41 | 0.04 | 4.58 |
| let-7c-5p | 29.6 | 1.0 | 0.61 | 1.64 | 30.1 | 0.88 | 0.09 | 9.08 |
| let-7d-5p | 29.6 | 1.0 | 0.55 | 1.83 | 29.6 | 1.26 | 0.56 | 2.86 |
| let-7e-5p | 25.9 | 1.0 | 0.81 | 1.23 | 26.8 | 0.73 | 0.17 | 3.08 |
| let-7g-5p | 27.6 | 1.0 | 0.58 | 1.72 | 29.5 | 0.38 | 0.13 | 1.09 |
| miR-100-5p | 26.7 | 1.0 | 0.47 | 2.11 | 29.3 | 0.23 | 0.01 | 4.21 |
| miR-101-3p | 29.8 | 1.0 | 0.40 | 2.48 | 30.8 | 0.72 | 0.35 | 1.47 |
| miR-106a-5p | 23.2 | 1.0 | 0.56 | 1.80 | 24.8 | 0.48 | 0.14 | 1.57 |
| miR-106b-3p | 30.9 | 1.0 | 0.08 | 12.47 | 33.2 | 0.21 | 0.01 | 7.21 |
| miR-106b-5p | 26.1 | 1.0 | 0.43 | 2.34 | 27.6 | 0.51 | 0.17 | 1.51 |
| miR-1180-3p | 30.8 | 1.0 | 0.30 | 3.36 | 31.6 | 0.60 | 0.15 | 2.36 |
| miR-1183 | 26.4 | 1.0 | 0.08 | 12.56 | 30.1 | 0.07 | 0.01 | 0.83 |
| miR-1208 | 30.0 | 1.0 | 0.38 | 2.63 | 31.3 | 0.42 | 0.09 | 2.03 |
| miR-1225-3p | 23.6 | 1.0 | 0.23 | 4.41 | 26.4 | 0.14 | 0.00 | 19.60 |
| miR-1227-3p | 28.3 | 1.0 | 0.45 | 2.23 | 29.6 | 0.41 | 0.06 | 2.85 |
| miR-1233-3p | 23.4 | 1.0 | 0.32 | 3.09 | 25.1 | 0.28 | 0.01 | 9.52 |
| miR-1247-5p | 29.3 | 1.0 | 0.00 | 295.36 | 22.2 | 130.42 | 0.43 | 39,287 |
| miR-1254 | 27.0 | 1.0 | 0.21 | 4.67 | 27.5 | 0.69 | 0.27 | 1.78 |
| miR-1255b-5p | 29.3 | 1.0 | 0.10 | 9.87 | 27.2 | 4.72 | 1.12 | 19.83 |
| miR-125a-3p | 32.2 | 1.0 | 0.04 | 22.80 | 30.7 | 3.83 | 0.52 | 27.98 |
| miR-125a-5p | 29.8 | 1.0 | 0.32 | 3.12 | 31.2 | 0.53 | 0.17 | 1.71 |
| miR-125b-1-3p | 32.1 | 1.0 | 0.26 | 3.83 | 33.8 | 0.31 | 0.12 | 0.85 |
| miR-125b-5p | 26.5 | 1.0 | 0.69 | 1.44 | 27.7 | 0.63 | 0.15 | 2.57 |
| miR-1260a | 25.0 | 1.0 | 0.47 | 2.15 | 26.6 | 0.33 | 0.01 | 7.26 |
| miR-1262 | 13.6 | 1.0 | 0.00 | 345.12 | 20.8 | 0.01 | 0.00 | 163.88 |
| miR-126-3p | 29.2 | 1.0 | 0.58 | 1.71 | 29.8 | 0.92 | 0.80 | 1.06 |
| miR-1267 | 28.6 | 1.0 | 0.16 | 6.26 | 29.6 | 0.50 | 0.09 | 2.77 |
| miR-1271-5p | 30.8 | 1.0 | 0.41 | 2.44 | 30.9 | 0.94 | 0.14 | 6.26 |
| miR-1274a | 23.4 | 1.0 | 0.50 | 1.98 | 24.7 | 0.41 | 0.09 | 1.88 |
| miR-1274b | 20.5 | 1.0 | 0.58 | 1.72 | 22.5 | 0.26 | 0.03 | 2.17 |
| miR-1275 | 24.0 | 1.0 | 0.38 | 2.66 | 26.1 | 0.23 | 0.00 | 21.06 |
| miR-128-3p | 27.5 | 1.0 | 0.36 | 2.77 | 28.4 | 0.74 | 0.30 | 1.81 |
| miR-1285-3p | 26.1 | 1.0 | 0.12 | 8.63 | 27.7 | 0.31 | 0.01 | 10.39 |
| miR-1290 | 25.6 | 1.0 | 0.50 | 2.02 | 25.0 | 1.56 | 0.71 | 3.41 |
| miR-1291 | 29.9 | 1.0 | 0.16 | 6.46 | 30.8 | 0.52 | 0.12 | 2.35 |
| miR-1303 | 26.8 | 1.0 | 0.32 | 3.10 | 28.5 | 0.30 | 0.04 | 2.25 |
| miR-130a-3p | 27.6 | 1.0 | 0.45 | 2.21 | 28.0 | 1.13 | 0.66 | 1.91 |
| miR-130b-3p | 27.6 | 1.0 | 0.41 | 2.47 | 27.0 | 2.08 | 0.99 | 4.35 |
| miR-132-3p | 25.9 | 1.0 | 0.34 | 2.94 | 27.7 | 0.40 | 0.03 | 5.10 |
| miR-133a-3p | 25.3 | 1.0 | 0.00 | 3,711.74 | 30.0 | 0.05 | 0.01 | 0.29 |
| miR-135a-5p | 31.8 | 1.0 | 0.47 | 2.11 | 33.6 | 0.45 | 0.03 | 6.62 |
| miR-135b-3p | 31.0 | 1.0 | 0.23 | 4.35 | 30.1 | 1.92 | 0.45 | 8.12 |
| miR-135b-5p | 28.3 | 1.0 | 0.77 | 1.29 | 29.8 | 0.53 | 0.11 | 2.52 |
| miR-138-5p | 30.6 | 1.0 | 0.32 | 3.12 | 31.2 | 0.85 | 0.27 | 2.73 |
| miR-139-5p | 30.3 | 1.0 | 0.36 | 2.78 | 33.3 | 0.20 | 0.04 | 1.08 |
| miR-140-3p | 28.2 | 1.0 | 0.33 | 3.06 | 29.6 | 0.48 | 0.07 | 3.25 |
| miR-141-3p | 26.3 | 1.0 | 0.36 | 2.76 | 24.2 | 6.28 | 0.02 | 2,169.60 |
| miR-142-3p | 25.5 | 1.0 | 0.02 | 52.76 | 26.9 | 0.52 | 0.00 | 60.08 |
| miR-142-5p | 27.5 | 1.0 | 0.52 | 1.93 | 28.6 | 0.63 | 0.23 | 1.72 |
| miR-143-3p | 26.8 | 1.0 | 0.29 | 3.41 | 26.9 | 1.23 | 0.43 | 3.48 |
| miR-145-3p | 29.9 | 1.0 | 0.18 | 5.45 | 30.3 | 0.78 | 0.21 | 2.90 |
| miR-145-5p | 22.9 | 1.0 | 0.34 | 2.98 | 25.5 | 0.24 | 0.03 | 1.90 |
| miR-146a-5p | 23.7 | 1.0 | 0.53 | 1.88 | 26.9 | 0.16 | 0.00 | 4.65 |
| miR-146b-5p | 25.4 | 1.0 | 0.13 | 7.91 | 27.2 | 0.42 | 0.21 | 0.85 |
| miR-148a-3p | 25.1 | 1.0 | 0.57 | 1.76 | 26.0 | 0.75 | 0.30 | 1.88 |
| miR-148b-3p | 28.6 | 1.0 | 0.36 | 2.78 | 30.1 | 0.50 | 0.27 | 0.95 |
| miR-149-5p | 26.6 | 1.0 | 0.22 | 4.61 | 27.3 | 0.82 | 0.34 | 1.94 |
| miR-150-5p | 27.6 | 1.0 | 0.47 | 2.12 | 27.5 | 1.47 | 0.54 | 4.02 |
| miR-151-3p | 27.8 | 1.0 | 0.36 | 2.80 | 29.1 | 0.40 | 0.11 | 1.50 |
| miR-152-3p | 28.2 | 1.0 | 0.67 | 1.49 | 29.0 | 0.82 | 0.27 | 2.51 |
| miR-155-5p | 29.4 | 1.0 | 0.54 | 1.86 | 28.8 | 2.15 | 1.05 | 4.41 |
| miR-15a-3p | 24.5 | 1.0 | 0.00 | 239,034 | 31.1 | 0.01 | 0.00 | 0.09 |
| miR-15b-5p | 26.2 | 1.0 | 0.53 | 1.88 | 27.3 | 0.66 | 0.28 | 1.56 |
| miR-16-5p | 22.3 | 1.0 | 0.37 | 2.68 | 23.5 | 0.61 | 0.20 | 1.85 |
| miR-17-5p | 23.3 | 1.0 | 0.52 | 1.90 | 24.9 | 0.46 | 0.15 | 1.45 |
| miR-181a-5p | 28.4 | 1.0 | 0.63 | 1.59 | 29.2 | 0.84 | 0.31 | 2.25 |
| miR-183-3p | 30.8 | 1.0 | 0.39 | 2.58 | 29.3 | 2.77 | 0.41 | 18.83 |
| miR-184 | 31.7 | 1.0 | 0.36 | 2.76 | 32.0 | 1.08 | 0.18 | 6.36 |
| miR-185-5p | 26.9 | 1.0 | 0.35 | 2.84 | 27.6 | 0.90 | 0.55 | 1.47 |
| miR-186-5p | 25.1 | 1.0 | 0.40 | 2.50 | 26.4 | 0.54 | 0.26 | 1.12 |
| miR-18a-5p | 29.0 | 1.0 | 0.14 | 7.07 | 29.9 | 0.70 | 0.17 | 2.92 |
| miR-190b | 11.6 | 1.0 | 0.03 | 33.98 | 21.9 | 0.00 | 0.00 | 1.16 |
| miR-191-3p | 30.9 | 1.0 | 0.26 | 3.82 | 29.1 | 3.41 | 0.70 | 16.53 |
| miR-191-5p | 23.3 | 1.0 | 0.33 | 3.04 | 25.5 | 0.32 | 0.11 | 0.94 |
| miR-192-5p | 28.8 | 1.0 | 0.52 | 1.92 | 29.5 | 0.87 | 0.48 | 1.61 |
| miR-193a-3p | 29.8 | 1.0 | 0.35 | 2.85 | 29.9 | 1.44 | 0.45 | 4.55 |
| miR-193a-5p | 26.2 | 1.0 | 0.44 | 2.28 | 26.1 | 1.54 | 0.67 | 3.58 |
| miR-193b-3p | 21.4 | 1.0 | 0.60 | 1.68 | 23.8 | 0.26 | 0.05 | 1.27 |
| miR-194-5p | 29.5 | 1.0 | 0.46 | 2.17 | 30.5 | 0.71 | 0.47 | 1.07 |
| miR-195-5p | 27.2 | 1.0 | 0.50 | 2.01 | 28.2 | 0.68 | 0.26 | 1.74 |
| miR-197-3p | 21.8 | 1.0 | 0.26 | 3.84 | 22.6 | 0.85 | 0.27 | 2.70 |
| miR-199a-3p | 27.4 | 1.0 | 0.29 | 3.42 | 29.3 | 0.37 | 0.02 | 6.35 |
| miR-19a-3p | 25.9 | 1.0 | 0.43 | 2.34 | 26.9 | 0.66 | 0.35 | 1.24 |
| miR-19b-1-5p | 27.5 | 1.0 | 0.01 | 67.90 | 24.2 | 9.58 | 0.00 | 24,122.31 |
| miR-19b-3p | 20.8 | 1.0 | 0.37 | 2.68 | 22.1 | 0.55 | 0.28 | 1.08 |
| miR-200a-3p | 26.8 | 1.0 | 0.11 | 9.05 | 28.8 | 0.36 | 0.01 | 14.92 |
| miR-200a-5p | 30.5 | 1.0 | 0.05 | 20.20 | 30.4 | 1.02 | 0.14 | 7.36 |
| miR-200b-3p | 26.6 | 1.0 | 0.02 | 43.17 | 26.8 | 1.20 | 0.11 | 13.16 |
| miR-200c-3p | 24.9 | 1.0 | 0.03 | 31.87 | 24.7 | 1.40 | 0.10 | 19.89 |
| miR-202-3p | 25.2 | 1.0 | 0.00 | 3,095.65 | 29.4 | 0.07 | 0.01 | 0.55 |
| miR-203a-3p | 20.1 | 1.0 | 0.63 | 1.59 | 22.5 | 0.27 | 0.01 | 11.40 |
| miR-204-5p | 29.2 | 1.0 | 0.45 | 2.22 | 28.8 | 1.83 | 0.28 | 11.99 |
| miR-205-5p | 24.1 | 1.0 | 0.27 | 3.66 | 25.7 | 0.49 | 0.08 | 2.86 |
| miR-206 | 32.0 | 1.0 | 0.08 | 12.28 | 31.7 | 1.26 | 0.25 | 6.41 |
| miR-20a-5p | 24.1 | 1.0 | 0.51 | 1.97 | 25.5 | 0.50 | 0.15 | 1.66 |
| miR-20b-5p | 27.4 | 1.0 | 0.58 | 1.72 | 29.2 | 0.41 | 0.10 | 1.68 |
| miR-210-3p | 23.2 | 1.0 | 0.13 | 7.66 | 24.6 | 0.55 | 0.03 | 9.31 |
| miR-211-5p | 28.3 | 1.0 | 0.31 | 3.23 | 26.9 | 3.51 | 0.40 | 31.16 |
| miR-212-3p | 28.3 | 1.0 | 0.62 | 1.62 | 28.3 | 1.37 | 0.48 | 3.89 |
| miR-21-5p | 22.6 | 1.0 | 0.53 | 1.87 | 22.8 | 1.19 | 0.62 | 2.27 |
| miR-218-5p | 29.7 | 1.0 | 0.19 | 5.29 | 28.0 | 4.15 | 1.82 | 9.47 |
| miR-221-3p | 25.1 | 1.0 | 0.53 | 1.88 | 28.1 | 0.16 | 0.00 | 4.60 |
| miR-222-3p | 22.5 | 1.0 | 0.60 | 1.66 | 23.8 | 0.58 | 0.25 | 1.36 |
| miR-222-5p | 30.6 | 1.0 | 0.44 | 2.26 | 29.4 | 2.35 | 0.57 | 9.57 |
| miR-223-3p | 15.2 | 1.0 | 0.36 | 2.74 | 16.8 | 0.46 | 0.12 | 1.76 |
| miR-223-5p | 24.6 | 1.0 | 0.36 | 2.75 | 26.6 | 0.25 | 0.08 | 0.77 |
| miR-22-3p | 24.5 | 1.0 | 0.37 | 2.68 | 24.8 | 1.13 | 0.31 | 4.13 |
| miR-224-5p | 26.2 | 1.0 | 0.58 | 1.73 | 27.6 | 0.53 | 0.07 | 4.32 |
| miR-22-5p | 28.5 | 1.0 | 0.23 | 4.39 | 29.8 | 0.42 | 0.03 | 5.18 |
| miR-24-3p | 19.8 | 1.0 | 0.53 | 1.88 | 21.3 | 0.51 | 0.10 | 2.60 |
| miR-25-3p | 25.2 | 1.0 | 0.44 | 2.29 | 25.5 | 1.11 | 0.44 | 2.80 |
| miR-26a-5p | 25.4 | 1.0 | 0.57 | 1.75 | 26.5 | 0.64 | 0.29 | 1.41 |
| miR-26b-5p | 27.4 | 1.0 | 0.33 | 3.04 | 28.6 | 0.60 | 0.22 | 1.65 |
| miR-27b-3p | 25.0 | 1.0 | 0.49 | 2.04 | 28.1 | 0.17 | 0.01 | 5.07 |
| miR-28-3p | 26.9 | 1.0 | 0.18 | 5.55 | 24.8 | 6.20 | 0.06 | 610.50 |
| miR-28-5p | 28.1 | 1.0 | 0.10 | 9.76 | 29.3 | 0.54 | 0.13 | 2.30 |
| miR-296-5p | 29.0 | 1.0 | 0.62 | 1.61 | 29.2 | 1.26 | 0.41 | 3.86 |
| miR-298 | 32.6 | 1.0 | 0.34 | 2.97 | 31.5 | 3.36 | 0.20 | 55.58 |
| miR-299-5p | 17.0 | 1.0 | 0.00 | 2,553 | 11.7 | 60.76 | 27.71 | 133.25 |
| miR-29a-3p | 24.2 | 1.0 | 0.39 | 2.58 | 25.6 | 0.51 | 0.08 | 3.17 |
| miR-29a-5p | 29.6 | 1.0 | 0.15 | 6.49 | 30.3 | 0.65 | 0.01 | 57.58 |
| miR-29b-3p | 30.4 | 1.0 | 0.82 | 1.21 | 27.5 | 10.33 | 0.00 | 202,876.83 |
| miR-29c-3p | 30.1 | 1.0 | 0.63 | 1.58 | 30.8 | 0.89 | 0.28 | 2.79 |
| miR-301a-3p | 28.5 | 1.0 | 0.36 | 2.77 | 30.6 | 0.32 | 0.10 | 1.01 |
| miR-30a-3p | 27.2 | 1.0 | 0.40 | 2.50 | 29.9 | 0.15 | 0.02 | 1.44 |
| miR-30a-5p | 24.8 | 1.0 | 0.32 | 3.16 | 27.0 | 0.21 | 0.07 | 0.66 |
| miR-30b-5p | 23.6 | 1.0 | 0.56 | 1.77 | 24.6 | 0.63 | 0.25 | 1.57 |
| miR-30c-5p | 23.2 | 1.0 | 0.43 | 2.33 | 26.7 | 0.13 | 0.00 | 7.79 |
| miR-30d-5p | 26.4 | 1.0 | 0.23 | 4.29 | 28.7 | 0.21 | 0.09 | 0.50 |
| miR-30e-3p | 26.7 | 1.0 | 0.55 | 1.81 | 28.9 | 0.21 | 0.07 | 0.65 |
| miR-31-3p | 29.1 | 1.0 | 0.01 | 80.43 | 24.2 | 33.06 | 0.00 | 10,242,902 |
| miR-31-5p | 26.5 | 1.0 | 0.68 | 1.46 | 26.5 | 1.30 | 0.79 | 2.14 |
| miR-320a | 25.0 | 1.0 | 0.50 | 2.00 | 25.0 | 1.40 | 0.68 | 2.89 |
| miR-320b | 31.5 | 1.0 | 0.06 | 16.61 | 30.6 | 1.98 | 0.52 | 7.44 |
| miR-323-3p | 32.1 | 1.0 | 0.19 | 5.20 | 31.2 | 2.56 | 0.86 | 7.63 |
| miR-324-3p | 28.7 | 1.0 | 0.54 | 1.87 | 29.2 | 0.97 | 0.56 | 1.67 |
| miR-324-5p | 28.0 | 1.0 | 0.67 | 1.50 | 28.6 | 0.93 | 0.35 | 2.47 |
| miR-32-5p | 31.1 | 1.0 | 0.15 | 6.73 | 32.2 | 0.62 | 0.04 | 9.46 |
| miR-328-3p | 25.0 | 1.0 | 0.60 | 1.65 | 25.3 | 1.17 | 0.54 | 2.56 |
| miR-330-3p | 30.3 | 1.0 | 0.22 | 4.58 | 33.1 | 0.19 | 0.04 | 0.96 |
| miR-331-3p | 26.2 | 1.0 | 0.57 | 1.76 | 26.7 | 0.99 | 0.61 | 1.61 |
| miR-331-5p | 29.1 | 1.0 | 0.39 | 2.54 | 28.9 | 1.65 | 0.66 | 4.13 |
| miR-335-3p | 30.6 | 1.0 | 0.28 | 3.59 | 30.9 | 0.78 | 0.24 | 2.53 |
| miR-335-5p | 29.1 | 1.0 | 0.33 | 3.01 | 29.4 | 1.15 | 0.75 | 1.75 |
| miR-338-3p | 28.0 | 1.0 | 0.14 | 7.01 | 29.6 | 0.46 | 0.18 | 1.19 |
| miR-339-3p | 27.6 | 1.0 | 0.63 | 1.59 | 29.9 | 0.30 | 0.01 | 6.46 |
| miR-33a-3p | 30.9 | 1.0 | 0.37 | 2.73 | 30.4 | 1.47 | 0.89 | 2.42 |
| miR-340-3p | 30.7 | 1.0 | 0.25 | 4.01 | 32.7 | 0.24 | 0.05 | 1.05 |
| miR-340-5p | 28.3 | 1.0 | 0.53 | 1.90 | 29.3 | 0.68 | 0.28 | 1.63 |
| miR-342-3p | 25.0 | 1.0 | 0.37 | 2.70 | 26.4 | 0.52 | 0.30 | 0.88 |
| miR-345-5p | 26.1 | 1.0 | 0.45 | 2.23 | 27.0 | 0.76 | 0.31 | 1.85 |
| miR-34a-3p | 28.8 | 1.0 | 0.33 | 3.02 | 30.0 | 0.43 | 0.16 | 1.16 |
| miR-34a-5p | 26.2 | 1.0 | 0.65 | 1.54 | 28.0 | 0.43 | 0.04 | 4.38 |
| miR-34b-3p | 25.5 | 1.0 | 0.25 | 3.92 | 26.6 | 0.45 | 0.06 | 3.39 |
| miR-34c-5p | 31.4 | 1.0 | 0.37 | 2.74 | 31.2 | 1.60 | 0.25 | 10.26 |
| miR-361-5p | 28.5 | 1.0 | 0.38 | 2.63 | 29.3 | 0.77 | 0.17 | 3.43 |
| miR-362-3p | 31.3 | 1.0 | 0.62 | 1.63 | 32.2 | 0.71 | 0.24 | 2.13 |
| miR-362-5p | 31.0 | 1.0 | 0.31 | 3.17 | 32.0 | 0.72 | 0.08 | 6.92 |
| miR-363-3p | 31.4 | 1.0 | 0.58 | 1.73 | 31.9 | 0.93 | 0.21 | 4.09 |
| miR-365-3p | 24.5 | 1.0 | 0.44 | 2.28 | 26.7 | 0.30 | 0.03 | 2.90 |
| miR-370-3p | 29.0 | 1.0 | 0.49 | 2.04 | 29.7 | 0.90 | 0.09 | 9.13 |
| miR-372-3p | 32.0 | 1.0 | 0.45 | 2.20 | 30.9 | 3.07 | 0.57 | 16.49 |
| miR-374a-5p | 28.0 | 1.0 | 0.62 | 1.62 | 29.1 | 0.60 | 0.19 | 1.90 |
| miR-375 | 21.2 | 1.0 | 0.60 | 1.68 | 22.7 | 0.50 | 0.08 | 3.16 |
| miR-376a-3p | 32.6 | 1.0 | 0.23 | 4.38 | 32.5 | 1.63 | 0.36 | 7.43 |
| miR-376c-3p | 31.1 | 1.0 | 0.47 | 2.12 | 33.7 | 0.17 | 0.08 | 0.32 |
| miR-378 | 26.6 | 1.0 | 0.38 | 2.60 | 28.0 | 0.37 | 0.10 | 1.45 |
| miR-378a-5p | 30.9 | 1.0 | 0.45 | 2.23 | 32.0 | 0.48 | 0.02 | 9.90 |
| miR-380-5p | 29.3 | 1.0 | 0.46 | 2.17 | 31.0 | 0.29 | 0.02 | 5.31 |
| miR-381-3p | 32.7 | 1.0 | 0.36 | 2.79 | 32.0 | 2.49 | 0.79 | 7.86 |
| miR-383-5p | 33.7 | 1.0 | 0.02 | 59.32 | 30.7 | 10.48 | 2.53 | 43.48 |
| miR-409-3p | 33.1 | 1.0 | 0.17 | 6.03 | 32.8 | 1.29 | 0.02 | 83.40 |
| miR-412-3p | 30.0 | 1.0 | 0.29 | 3.44 | 27.4 | 9.40 | 5.86 | 15.10 |
| miR-422a | 31.3 | 1.0 | 0.43 | 2.30 | 30.9 | 1.95 | 1.00 | 3.78 |
| miR-423-5p | 27.0 | 1.0 | 0.26 | 3.80 | 27.5 | 1.02 | 0.17 | 6.12 |
| miR-424-3p | 28.3 | 1.0 | 0.23 | 4.38 | 30.1 | 0.31 | 0.04 | 2.66 |
| miR-424-5p | 32.0 | 1.0 | 0.54 | 1.85 | 31.7 | 1.62 | 1.46 | 1.79 |
| miR-425-3p | 29.8 | 1.0 | 0.64 | 1.56 | 29.6 | 1.15 | 0.60 | 2.19 |
| miR-425-5p | 23.9 | 1.0 | 0.45 | 2.24 | 24.3 | 1.05 | 0.51 | 2.15 |
| miR-429 | 27.8 | 1.0 | 0.89 | 1.13 | 29.4 | 0.46 | 0.10 | 2.11 |
| miR-433-3p | 29.6 | 1.0 | 0.55 | 1.81 | 30.4 | 0.80 | 0.25 | 2.63 |
| miR-449a | 31.5 | 1.0 | 0.34 | 2.95 | 31.9 | 1.22 | 0.36 | 4.10 |
| miR-449b-5p | 33.1 | 1.0 | 0.03 | 32.19 | 33.3 | 1.40 | 0.04 | 49.01 |
| miR-450a-5p | 31.0 | 1.0 | 0.55 | 1.83 | 32.1 | 0.59 | 0.01 | 30.90 |
| miR-452-5p | 29.1 | 1.0 | 0.63 | 1.60 | 29.9 | 0.84 | 0.29 | 2.47 |
| miR-454-3p | 29.6 | 1.0 | 0.40 | 2.47 | 31.4 | 0.41 | 0.13 | 1.24 |
| miR-483-5p | 29.7 | 1.0 | 0.29 | 3.49 | 30.4 | 0.84 | 0.07 | 10.41 |
| miR-484 | 20.5 | 1.0 | 0.54 | 1.86 | 22.1 | 0.44 | 0.23 | 0.85 |
| miR-485-3p | 31.2 | 1.0 | 0.42 | 2.38 | 30.3 | 3.05 | 0.20 | 46.14 |
| miR-486-3p | 27.5 | 1.0 | 0.00 | 856.57 | 31.7 | 0.08 | 0.00 | 2.99 |
| miR-486-5p | 25.5 | 1.0 | 0.00 | 7,021.08 | 25.9 | 1.14 | 0.00 | 5,223.25 |
| miR-487b-3p | 32.0 | 1.0 | 0.04 | 25.69 | 28.8 | 14.69 | 0.03 | 7,714.66 |
| miR-489-3p | 34.9 | 1.0 | 0.04 | 27.79 | 30.4 | 35.07 | 17.47 | 70.41 |
| miR-500a-5p | 31.1 | 1.0 | 0.07 | 13.54 | 30.2 | 2.13 | 0.44 | 10.42 |
| miR-502-3p | 31.0 | 1.0 | 0.47 | 2.13 | 31.3 | 1.04 | 0.27 | 4.02 |
| miR-502-5p | 31.4 | 1.0 | 0.31 | 3.24 | 30.3 | 2.86 | 0.54 | 15.23 |
| miR-505-3p | 28.7 | 1.0 | 0.33 | 3.06 | 29.4 | 0.76 | 0.15 | 3.74 |
| miR-509-5p | 30.9 | 1.0 | 0.22 | 4.45 | 30.1 | 2.44 | 0.19 | 30.54 |
| miR-511-5p | 31.1 | 1.0 | 0.13 | 7.61 | 29.3 | 5.01 | 1.80 | 13.92 |
| miR-512-3p | 29.8 | 1.0 | 0.35 | 2.82 | 28.1 | 5.13 | 2.05 | 12.85 |
| miR-516-3p | 30.9 | 1.0 | 0.16 | 6.47 | 30.0 | 1.79 | 0.36 | 8.87 |
| miR-517a-3p | 31.0 | 1.0 | 0.43 | 2.31 | 33.0 | 0.34 | 0.01 | 10.42 |
| miR-517c-3p | 31.2 | 1.0 | 0.52 | 1.93 | 32.7 | 0.51 | 0.07 | 3.62 |
| miR-518b | 31.6 | 1.0 | 0.04 | 25.15 | 29.8 | 5.16 | 0.63 | 42.59 |
| miR-518d-5p | 27.0 | 1.0 | 0.00 | 2,698.54 | 29.1 | 0.50 | 0.04 | 6.55 |
| miR-518f-3p | 30.0 | 1.0 | 0.06 | 15.52 | 23.1 | 216.11 | 0.00 | 69,561,245,696 |
| miR-519e-3p | 30.7 | 1.0 | 0.04 | 26.01 | 30.7 | 1.81 | 0.55 | 5.97 |
| miR-532-5p | 28.5 | 1.0 | 0.03 | 30.75 | 30.4 | 0.32 | 0.00 | 27.25 |
| miR-541-5p | 30.4 | 1.0 | 0.32 | 3.08 | 32.0 | 0.30 | 0.05 | 1.76 |
| miR-545-3p | 31.7 | 1.0 | 0.44 | 2.26 | 29.4 | 7.53 | 1.83 | 30.90 |
| miR-545-5p | 25.3 | 1.0 | 0.00 | 1,109.08 | 21.2 | 16.74 | 0.06 | 4,612.45 |
| miR-548b-5p | 27.8 | 1.0 | 0.31 | 3.26 | 27.3 | 2.07 | 0.21 | 20.40 |
| miR-548c-5p | 27.8 | 1.0 | 0.22 | 4.47 | 27.0 | 2.35 | 0.26 | 21.17 |
| miR-548d-5p | 30.9 | 1.0 | 0.08 | 12.97 | 29.6 | 3.41 | 0.21 | 55.27 |
| miR-566 | 24.9 | 1.0 | 0.27 | 3.66 | 26.5 | 0.34 | 0.02 | 5.79 |
| miR-570-3p | 33.1 | 1.0 | 0.15 | 6.81 | 30.9 | 7.41 | 1.06 | 51.96 |
| miR-571 | 26.2 | 1.0 | 0.37 | 2.74 | 27.9 | 0.30 | 0.07 | 1.20 |
| miR-572 | 29.5 | 1.0 | 0.43 | 2.32 | 30.7 | 0.44 | 0.14 | 1.36 |
| miR-574-3p | 24.3 | 1.0 | 0.79 | 1.26 | 25.1 | 0.83 | 0.44 | 1.56 |
| miR-576-3p | 30.0 | 1.0 | 0.50 | 1.99 | 30.8 | 0.91 | 0.47 | 1.79 |
| miR-579-3p | 30.4 | 1.0 | 0.42 | 2.39 | 31.5 | 0.68 | 0.36 | 1.27 |
| miR-582-3p | 31.6 | 1.0 | 0.10 | 10.22 | 32.9 | 0.63 | 0.06 | 6.49 |
| miR-584-5p | 27.7 | 1.0 | 0.20 | 4.89 | 28.3 | 0.64 | 0.11 | 3.67 |
| miR-589-3p | 30.4 | 1.0 | 0.15 | 6.47 | 30.8 | 0.78 | 0.10 | 5.89 |
| miR-590-3p | 31.7 | 1.0 | 0.57 | 1.75 | 32.5 | 0.56 | 0.17 | 1.83 |
| miR-590-5p | 26.5 | 1.0 | 0.54 | 1.87 | 27.4 | 0.78 | 0.37 | 1.65 |
| miR-597-5p | 30.5 | 1.0 | 0.32 | 3.07 | 29.1 | 3.62 | 1.83 | 7.14 |
| miR-598-3p | 24.6 | 1.0 | 0.08 | 12.48 | 24.2 | 1.83 | 0.00 | 1,430.02 |
| miR-601 | 31.0 | 1.0 | 0.43 | 2.33 | 31.7 | 0.61 | 0.47 | 0.78 |
| miR-603 | 32.2 | 1.0 | 0.38 | 2.65 | 31.1 | 2.36 | 1.30 | 4.28 |
| miR-604 | 31.3 | 1.0 | 0.29 | 3.40 | 28.7 | 6.62 | 0.33 | 131.71 |
| miR-605-5p | 27.4 | 1.0 | 0.54 | 1.84 | 27.2 | 1.09 | 0.15 | 7.67 |
| miR-616-3p | 30.4 | 1.0 | 0.11 | 9.37 | 30.1 | 2.11 | 0.06 | 75.56 |
| miR-616-5p | 30.4 | 1.0 | 0.82 | 1.23 | 31.5 | 0.50 | 0.09 | 2.89 |
| miR-618 | 30.4 | 1.0 | 0.56 | 1.79 | 30.5 | 1.21 | 0.16 | 8.91 |
| miR-623 | 27.6 | 1.0 | 0.09 | 11.47 | 28.4 | 0.55 | 0.03 | 11.84 |
| miR-625-3p | 27.9 | 1.0 | 0.47 | 2.14 | 29.7 | 0.27 | 0.01 | 5.15 |
| miR-625-5p | 30.5 | 1.0 | 0.39 | 2.56 | 33.0 | 0.28 | 0.11 | 0.72 |
| miR-627-5p | 18.7 | 1.0 | 0.00 | 2,153.82 | 18.0 | 1.99 | 0.00 | 13,902,729 |
| miR-628-5p | 32.0 | 1.0 | 0.22 | 4.60 | 26.1 | 87.56 | 0.05 | 170,413.844 |
| miR-629-5p | 28.9 | 1.0 | 0.54 | 1.84 | 29.8 | 0.52 | 0.22 | 1.24 |
| miR-629-5p | 29.0 | 1.0 | 0.50 | 2.01 | 31.3 | 0.27 | 0.01 | 13.01 |
| miR-630 | 32.4 | 1.0 | 0.09 | 11.39 | 31.0 | 2.46 | 1.18 | 5.15 |
| miR-636 | 26.1 | 1.0 | 0.01 | 97.45 | 26.1 | 1.24 | 0.01 | 184.53 |
| miR-638 | 28.0 | 1.0 | 0.41 | 2.42 | 29.5 | 0.35 | 0.04 | 3.38 |
| miR-642a-5p | 30.9 | 1.0 | 0.24 | 4.12 | 30.0 | 2.37 | 0.95 | 5.92 |
| miR-650 | 27.7 | 1.0 | 0.28 | 3.61 | 27.7 | 0.99 | 0.15 | 6.57 |
| miR-652-3p | 27.2 | 1.0 | 0.07 | 15.14 | 29.5 | 0.25 | 0.02 | 3.60 |
| miR-660-5p | 26.2 | 1.0 | 0.39 | 2.56 | 27.8 | 0.48 | 0.05 | 4.72 |
| miR-661 | 25.5 | 1.0 | 0.28 | 3.59 | 26.1 | 0.63 | 0.32 | 1.23 |
| miR-668-3p | 30.3 | 1.0 | 0.01 | 77.34 | 30.0 | 1.18 | 0.41 | 3.42 |
| miR-671-3p | 29.7 | 1.0 | 0.22 | 4.43 | 30.2 | 0.94 | 0.29 | 3.10 |
| miR-708-5p | 30.0 | 1.0 | 0.35 | 2.86 | 29.8 | 1.65 | 0.36 | 7.61 |
| miR-720 | 22.1 | 1.0 | 0.68 | 1.47 | 23.9 | 0.29 | 0.09 | 0.96 |
| miR-744-3p | 29.5 | 1.0 | 0.17 | 5.85 | 29.5 | 0.94 | 0.14 | 6.10 |
| miR-744-5p | 28.7 | 1.0 | 0.47 | 2.14 | 29.4 | 0.80 | 0.15 | 4.30 |
| miR-769-5p | 27.7 | 1.0 | 0.35 | 2.86 | 28.6 | 0.53 | 0.10 | 2.69 |
| miR-770-5p | 31.0 | 1.0 | 0.05 | 19.43 | 30.8 | 1.12 | 0.15 | 8.16 |
| miR-873-5p | 30.2 | 1.0 | 0.39 | 2.55 | 29.3 | 3.01 | 1.29 | 7.01 |
| miR-885-5p | 26.8 | 1.0 | 0.75 | 1.34 | 28.2 | 0.53 | 0.22 | 1.29 |
| miR-886-3p | 29.9 | 1.0 | 0.47 | 2.15 | 30.1 | 1.20 | 0.66 | 2.17 |
| miR-886-5p | 30.7 | 1.0 | 0.34 | 2.90 | 31.1 | 1.08 | 0.46 | 2.54 |
| miR-892b | 29.6 | 1.0 | 0.16 | 6.43 | 30.4 | 0.58 | 0.14 | 2.41 |
| miR-92a-3p | 23.5 | 1.0 | 0.39 | 2.58 | 24.5 | 0.75 | 0.23 | 2.42 |
| miR-93-3p | 27.7 | 1.0 | 0.54 | 1.85 | 29.0 | 0.39 | 0.15 | 1.05 |
| miR-939-5p | 25.9 | 1.0 | 0.27 | 3.75 | 27.3 | 0.35 | 0.05 | 2.35 |
| miR-9-3p | 31.1 | 1.0 | 0.28 | 3.61 | 31.7 | 0.67 | 0.23 | 1.93 |
| miR-943 | 28.5 | 1.0 | 0.18 | 5.64 | 30.6 | 0.24 | 0.05 | 1.26 |
| miR-95-3p | 27.9 | 1.0 | 0.49 | 2.05 | 29.5 | 0.44 | 0.12 | 1.70 |
| miR-9-5p | 29.0 | 1.0 | 0.35 | 2.88 | 29.3 | 1.20 | 0.71 | 2.02 |
| miR-99a-5p | 27.0 | 1.0 | 0.68 | 1.48 | 29.1 | 0.32 | 0.01 | 7.88 |
| miR-99b-3p | 31.9 | 1.0 | 0.25 | 3.99 | 31.0 | 1.88 | 0.37 | 9.48 |
| miR-99b-5p | 27.6 | 1.0 | 0.69 | 1.45 | 28.5 | 0.77 | 0.40 | 1.48 |
| rno-miR-7-1-3p | 26.7 | 1.0 | 0.29 | 3.43 | 27.3 | 0.65 | 0.11 | 3.73 |
| U6 snRNA | 18.8 | 1.0 | 0.48 | 2.09 | 20.4 | 0.38 | 0.06 | 2.45 |

**Table S2: miRNAs expressed in both controls and OSCC patients.**
